# Supplementary material for: Higher order microfibre modes for dielectric particle trapping and propulsion
Source: Sci Rep. 2015 Mar 13;5:9077. doi: 10.1038/srep09077 (PMC4357993; doi:10.1038/srep09077)
Supplement: Supplementary Information — supplementary infomation [file srep09077-s1.pdf]

Higher order microfibre modes for dielectric particle trapping and propulsion

Aili Maimaiti[1,2], Viet Giang Truong[1], Marios Sergides[1], Ivan Gusachenko[1]
& Síle Nic Chormaic*[1]
[1]*Light-Matter Interactions Unit, OIST Graduate University, Onna, Okinawa 904-0495, Japan,*
[2]*Physics Department, University College Cork, Cork, Ireland*
**sile.nicchormaic@oist.jp*

LP_{11} mode transmission during tapering process

To address the LP_{11} higher order modes (HOM) propagating in a microfibre, the transmission spectrum of the HOMs was recorded at the fibre output using a photodiode and a digital oscilloscope during the tapering process. Figure 1 shows the transmission in respect to the pull length. Here, the oscillation of power was mainly due to interference between guided HOMs and symmetric higher order cladding modes. As the fibre was tapered down to each mode cut-off diameter, the transmitted power droped substantially. Coupled HE_{21} modes have the same mode cut-off and reach mode drop first. The TE_{01} and TM_{01} modes have their mode cut-off slightly later (i.e. for a slightly smaller waist)[1]. The power portion of HE_{21} modes was around 40% and 43% for the TE_{01} and TM_{01} modes.

If the fibre is tapered down until just before it? breaks, after the single mode cut-off there was only residual FM propagating in the fibre. The small percentage of the unexpected fundamental mode was generated due to the quality of the LG_{01} beam and the coupling efficiency to the fibre. Subtracting this portion of the fundamental mode from the total power input, the proportion of initially generated HOMs in the untapered fibre was approximated to be 98%.

In our experiments the fibre pulling process was stopped at a pull length of 75 mm (vertical dashed line) that corresponded to the waist diameter of 2 µm.

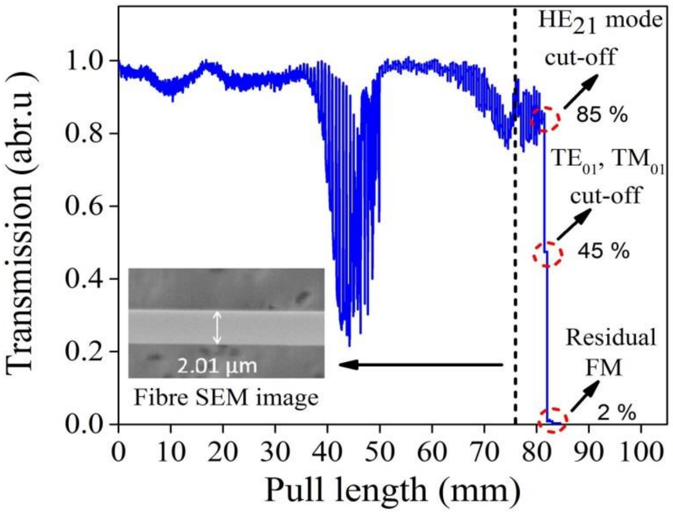

Supplementary Figure S1| Tapered fibre transmission loss at the corresponding mode cut-offs for each mode of the LP_{11} set. Here, the intensity drops due to the loss of the HE_{21} modes and the TE_{01} and TM_{01} modes. The inset is a SEM image of the 2 μm fibre.

Trapping and propulsion

We prepared a dilute particle solution with a particle/water volume ratio smaller than 1:10 000 to avoid the trapping of random particles by the fibre's evanescent field. Initially, the microfibre was positioned in the solution while the trapping beam was off. As the optical tweezers was switched on, the focused trapping beam could pick up a particle anywhere in the solution. The particle was then approached to the fibre. When the particle was released from the tweezers, it was propelled by the evanescent field of the fibre. The experiment was performed for a range of powers. The experimental procedure is shown in Fig. 2 below.

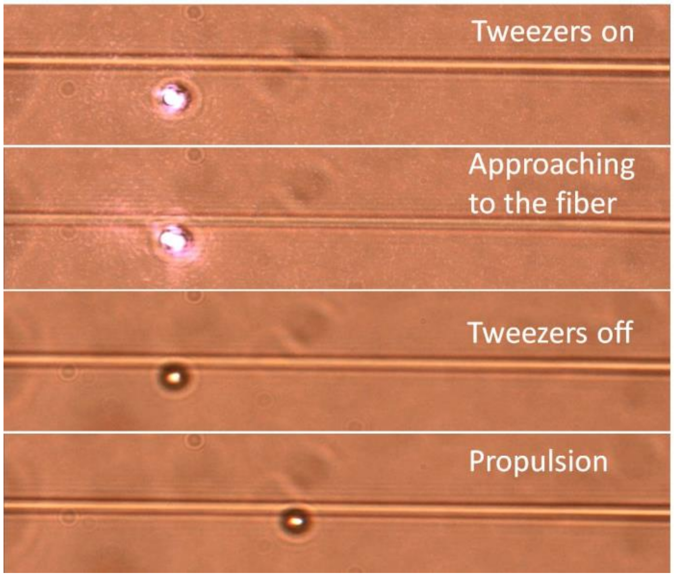

Supplementary Figure S2| Diagram for trapping-propelling experiment. (a-b) A 3 μm polystyrene sphere is trapped by the optical tweezers and approached to the fibre. (c-d) The particle is released from the optical tweezers trap and is propelled by the evanescent field of the fibre.

References

1. Frawley, M. C., Petcu-Colan, A., Truong, V. G. & Nic Chormaic, S. Higher order mode propagation in an optical nanofiber. *Opt. Commun.* **285,** 4648–4654 (2012).
